# Supplementary material for: Neural dynamics of visual ambiguity resolution by perceptual prior
Source: eLife. 2019 Mar 7;8:e41861. doi: 10.7554/eLife.41861 (PMC6415935; doi:10.7554/eLife.41861)
Supplement: Transparent reporting form [file elife-41861-transrepform.docx]

***eLife’s* transparent reporting form**

We encourage authors to provide detailed information *within their submission* to facilitate the interpretation and replication of experiments. Authors can upload supporting documentation to indicate the use of appropriate reporting guidelines for health-related research (see [EQUATOR Network](http://www.equator-network.org/%20)), life science research (see the [BioSharing Information Resource](https://biosharing.org/)), or the [ARRIVE guidelines](http://www.plosbiology.org/article/info:doi/10.1371/journal.pbio.1000412) for reporting work involving animal research. Where applicable, authors should refer to any relevant reporting standards documents in this form.

If you have any questions, please consult our Journal Policies and/or contact us: [editorial@elifesciences.org](mailto:editorial@elifesciences.org).

**Sample-size estimation**

- You should state whether an appropriate sample size was computed when the study was being designed
- You should state the statistical method of sample size computation and any required assumptions
- If no explicit power analysis was used, you should describe how you decided what sample (replicate) size (number) to use

Please outline where this information can be found within the submission (e.g., sections or figure legends), or explain why this information doesn’t apply to your submission:

Information about sample-size estimation can be found in the METHODS: Subjects section. The sample size was chosen based on previous MEG and 7T fMRI studies on perceptual / cognitive functions (Carlson et al., J Vis 2013; Li et al., J Neurosci 2014; Salti et al., 2015; Wardle et al., Neuroimage 2016). Our MEG sample size (n=23) was comparable to or larger than aforementioned studies (range 5-20). After excluding subjects with excessive motion, the final sample size was n=18.

Our additional fMRI dataset sample size (n=23) was larger than most 7T fMRI studies (range 5-15) (Emmerling et al., Neuroimage 2016; Sengupta et al., Neuroimage 2017; Suthana et al., J Cogn Neurosci 2015; De Martino et al., Nat Comm 2012; Klein et al., Neuron 2014; Yang et al., Neuroimage 2014). After excluding subjects with excessive motion, the final sample size was n=19.

**Replicates**

- You should report how often each experiment was performed
- You should include a definition of biological versus technical replication
- The data obtained should be provided and sufficient information should be provided to indicate the number of independent biological and/or technical replicates
- If you encountered any outliers, you should describe how these were handled
- Criteria for exclusion/inclusion of data should be clearly stated
- High-throughput sequence data should be uploaded before submission, with a private link for reviewers provided (these are available from both GEO and ArrayExpress)

Please outline where this information can be found within the submission (e.g., sections or figure legends), or explain why this information doesn’t apply to your submission:

Information about replicates and data exclusion can be found in METHODS: Subjects section. Out experiment was performed in n = 23 human participants, which constitutes a biological replicate. 5 subjects were excluded due to excessive head motion in the scanner.

**Statistical reporting**

- Statistical analysis methods should be described and justified
- Raw data should be presented in figures whenever informative to do so (typically when N per group is less than 10)
- For each experiment, you should identify the statistical tests used, exact values of N, definitions of center, methods of multiple test correction, and dispersion and precision measures (e.g., mean, median, SD, SEM, confidence intervals; and, for the major substantive results, a measure of effect size (e.g., Pearson's r, Cohen's d)
- Report exact p-values wherever possible alongside the summary statistics and 95% confidence intervals. These should be reported for all key questions and not only when the p-value is less than 0.05.

Please outline where this information can be found within the submission (e.g., sections or figure legends), or explain why this information doesn’t apply to your submission:

Details about our statistical analyses are included in the METHODS section as well as corresponding RESULTS section and figure legends.

Detailed statistics related to behavioral analyses (Fig. 2), including sample size and effect sizes, are included in RESULTS.

Decoding, RSA, and model-based data fusion analyses (Figs. 3-6) were performed at the population level, correcting for multiple comparisons across time points using nonparametric cluster-based permutation tests (Maris & Oostenveld, 2009). These are current and validated methods of multivariate pattern analyses for neuroimaging data (Pereira et al., Neuroimage 2009; Haxby et al., Annu Rev Neurosci 2014; Grootswagers et al., J Cog Neurosci 2017; Guggenmos et al., Neuroimage 2018; Hebart et al., eLife 2018). For details, see METHODS: Cluster-based permutation tests for multivariate pattern decoding. Reporting effect sizes for decoding, RSA, and model-based data fusion analyses is not informative beyond the results presented that are inherent to these analyses (Hebart & Baker, Neuroimage 2017).

(For large datasets, or papers with a very large number of statistical tests, you may upload a single table file with tests, Ns, etc., with reference to sections in the manuscript.)

**Group allocation**

- Indicate how samples were allocated into experimental groups (in the case of clinical studies, please specify allocation to treatment method); if randomization was used, please also state if restricted randomization was applied
- Indicate if masking was used during group allocation, data collection and/or data analysis

Please outline where this information can be found within the submission (e.g., sections or figure legends), or explain why this information doesn’t apply to your submission:

This information can be found in METHODS: Task paradigm. The fMRI experimental group completed an identical task (Gonzalez-Garcia, C. et al., eLife 2018). All participants were exposed to all combinations of our experimental factors. The order of Mooney image presentation was randomized across subjects. Importantly, all subjects participating in the MEG experiment were novel to these Mooney images. Thus, these subjects represent a population distinct from the population that participated in the fMRI experiment.

**Additional data files (“source data”)**

- We encourage you to upload relevant additional data files, such as numerical data that are represented as a graph in a figure, or as a summary table
- Where provided, these should be in the most useful format, and they can be uploaded as “Source data” files linked to a main figure or table
- Include model definition files including the full list of parameters used
- Include code used for data analysis (e.g., R, MatLab)
- Avoid stating that data files are “available upon request”

Please indicate the figures or tables for which source data files have been provided:

Source data and code for reproducing the analyses shown in Figs. 4-6 are provided.
